# Men's health care: concept analysis*

**DOI:** 10.17533/udea.iee.v41n1e14

**Published:** 2023-03-14

**Authors:** Anderson Reis de Sousa, Nuno Damácio de Carvalho Félix, Richardson Augusto Rosendo da Silva, Evanilda Souza de Santana Carvalho, Álvaro Pereira

**Affiliations:** 1 Nurse, PhD. Professor. Email: anderson.sousa@ufba.br. Graduate Program in Nursing and Health, School of Nursing, Federal University of Bahia. Salvador, BA, Brazil. Universidade Federal da Bahia School of Nursing Federal University of Bahia Salvador BA Brazil anderson.sousa@ufba.br; 2 Nurse, PhD. Professor. Federal University of Recôncavo da Bahia. Santo Antônio de Jesus, BA, Brazil. Email: nunofelix@ufrb.edu.br Universidade Federal do Recôncavo da Bahia Federal University of Recôncavo da Bahia Santo Antônio de Jesus BA Brazil nunofelix@ufrb.edu.br; 3 Nurse, PhD. Professor. Federal University of Rio Grande do Norte. Rio Grande do Norte, Brazil. Email: rirosendo@hotmail.com Universidade Federal do Rio Grande do Norte Federal University of Rio Grande do Norte Rio Grande do Norte Brazil rirosendo@hotmail.com; 4 Nurse, PhD. Professor. State University of Feira de Santana, Feira de Santana, BA, Brazil. Email: evasscarvalho@uefs.br Universidade Estadual de Feira de Santana State University of Feira de Santana Feira de Santana BA Brazil evasscarvalho@uefs.br; 5 Nurse, PhD. Professor. Graduate Program in Nursing and Health, School of Nursing, Federal University of Bahia. Salvador, BA, Brazil. Email: alvaro_pereira_ba@yahoo.com.br Universidade Federal da Bahia School of Nursing Federal University of Bahia Salvador BA Brazil alvaro_pereira_ba@yahoo.com.br

**Keywords:** man, men´s health, masculinity, nursing, hombres, salud del hombre, masculinidad, enfermería, homem, saúde do homem, empatia, masculinidade, enfermagem

## Abstract

**Objective.:**

To analyze the concept of men's health care and identify its essential, antecedent and consequent attributes in the health context.

**Methods.:**

This is a concept analysis structured in the theoretical-methodological framework of the Walker and Avant Model. An integrative review was carried out between May and July 2020, using keywords and descriptors: Men's Care and Health.

**Results.:**

The concept of men's health care is structured by 240 attributes, 14 categories, 82 antecedents and 159 consequents, from the selection of 26 published manuscripts. The design was evidenced from the dimensions: Intrapersonal, psychological and behavioral related to masculinities, interpersonal, organizational and structural, ecological, ethnoracial, cross-cultural and transpersonal.

**Conclusion.:**

The concept of men's health care revealed the male specificities regarding the recognition of the place of health care and the daily exercise in the lived experience.

## Resumen

**Objetivo.:**

Analizar el concepto de cuidado de la salud de los hombres e identificar sus atributos esenciales, antecedentes y consecuencias en el contexto sanitario.

**Métodos.:**

Se trata de un análisis conceptual estructurado en el marco teórico y metodológico del Modelo Walker y Avant. Se realizó una revisión integradora, mediante la utilización de palabras clave y descriptoras: Cuidado y Salud del Hombre.

**Resultados.:**

El concepto de cuidado de la salud de los hombres está estructurado por 240 atributos, 14 categorías, 82 antecedentes y 159 consecuencias, originados en los 26 manuscritos publicados seleccionados. Se evidenció el delineamiento a partir de las dimensiones: Intrapersonal, psicológicas y conductuales relacionadas con las masculinidades, interpersonal, organizacional y estructural, ecológica, étnico-racial, transcultural y transpesonales.

**Conclusión.:**

El concepto de cuidado de la salud de los hombres reveló las especificidades masculinas en cuanto al reconocimiento del lugar del cuidado de la salud y a su ejercicio cotidiano en la experiencia vivida.

## Resumo

**Objetivo.:**

Analisar o conceito do cuidado de saúde de homens e identificar respectivos atributos essenciais, antecedentes e consequentes no contexto sanitário.

**Métodos.:**

Se trata de uma análise de conceito estruturado no referencial teórico-metodológico do Modelo de Walker e Avant. Se realizou uma revisão integrativa entre o mês de maio a julho de 2020, mediante a utilização de palavras chaves e descritores: Cuidado e Saúde do Homem.

**Resultados.:**

O conceito de cuidado de saúde de homens está estruturado por 240 atributos, 14 categorias, 82 antecedentes e 159 consequentes, oriundos da seleção de 26 manuscritos publicados. Se evidenciou o delineamento a partir das dimensões: Intrapessoais, psicológicas e comportamentais relativas às masculinidades, interpessoais, organizacionais e estruturais, ecológicas, etnicoraciais, transculturais e transpessoais.

**Conclusão.:**

O conceito de cuidado de saúde de homens revelou as especificidades masculinas quanto ao reconhecimento do lugar do cuidado de saúde e o seu exercício cotidiano na experiência vivida.

## Introduction

The field of studies in the area of men's health has grown recently in the world in the last ten years. Most of the scientific production is directed to the hegemonic masculinity model, centered on the profile of white and heterosexual men, in which other peripheral models of masculinities that include black, poor men, residents in traditional communities and minority sexual and/or gender groups, are little present.^1^^-^^3^ Studies about men's health care are few and focused on injuries and diseases prevalent in the male population.^4^^-^^7^ When analyzing the advances in the field of male health, the emergence of public health policies, the development of programs, actions and technologies is noted^8^^-^^10^; however, conceptual gaps are identified, which impacts the advancement of knowledge about care directed to this public. Regarding the relevance and pertinence of the analysis and/or development of concept, especially in the field of health, the significant contribution to the addition and provision of responses to gaps under the phenomenon of male health care existing in the knowledge already produced stands out.^11^ Thus, with regard to the area of men's health, the development of a concept allows to instrumentalize and operationalize the focal, strategic and programmatic actions in health that involve this context. As a contribution, we can see the identification of the real needs of male health; qualified and resolute care planning; provocation of reflections by health professionals through the appropriation of the concept; and accommodation to the formulation and implementation of public policies. 

When performing a search in global databases such as the Medical Literature Analysis and Retrieval System Online - MEDLINE/ PubMed, the absence of studies related to the health care of men was identified, as well as the development of defined concepts, in which only the presence of isolated terms and/or uniterms was recognized. In this scenario, the apprehension of the available findings justified the decision to contribute to the development of the concept “men's health care”. Given the above, this study aimed to analyze the concept of health care for men and identify essential, antecedent and consequent attributes in the context of health.

## Methods

This is a concept analysis based on the theoretical-methodological model of Walker and Avant, whose composition is systematized in eight stages, namely: 1. Selection of the concept; 2. Delimitation of the objectives of the analysis; 3. Identification of the use of the concept in the literature; 4. Determination of essential attributes; 5. Identification of the model case; 6. Identification of contrary cases; 7. Identification of the background and consequences of the concept under analysis and 8. Definition of the empirical references of the developed concept.^12^ Conceptual analysis and development is understood as the careful process of examining the basic elements that make up a concept, with a focus on achieving the recognition of distinctions of similarities and differences.^13^ In this study, for the development of the concept of health care for men, it was taken into account that it emerges and addresses the context of health practice, is located in the need to deepen and understand the phenomenon of care, in addition to guiding its use in daily professional life.^14^

As proposed by Walker and Avant in the first stage, it was defined as a question for the development of the concept: How can men's health care be conceptualized? Thus, we adopted the inclusion criteria of the reviewed studies to achieve development, namely: having been produced in the area of health sciences, social sciences and humanities; presenting health care involving the male public as the centrality of the object/concept and composing relevant findings for the development of the concept in the health field. In the second stage, the objective was defined, followed by the third and fourth stage, which consisted of the theoretical exploration of the conceptual attributes. An integrative review was carried out on the elements that make up men's health care, in order to investigate the existence of theorized concepts, employment, frequency of appearance, adequacy, correlation and the definition of these in the literature. In order to compose the textual corpus of the study, the stages proposed by Mendes, Silveira and Galvão were adopted, namely: 1. Definition of the review question; 2. Search and selection of primary studies; 3. Extraction of data from primary studies; 4. Critical evaluation of primary studies; 5. Summary of the review results; 6. Presentation of the review.^15^

The questions adopted for data extraction in the literature were defined through the PICo strategy, whose acronym means: Population/Problem: Man; Interest: Essential, antecedent and consequent attributes; Context: Health care,^16^ namely: What theoretical and operational aspects can compose the concept of men's health care? How is men's health care defined in the studies analyzed? What elements can precede the emergence of this concept, and what can be identified as consequences? The selected texts met the inclusion criteria: primary studies, conducted with the male population, whose title had the terms care and related terms - care, self-care, care practice and others, health, men/man and/or male, without time frame, English, Spanish and Portuguese. Theses, dissertations, technical reports, health policies, opinion articles letters to the editor, experience reports, previous notes, duplicate studies, available for free access only the abstract, systematic review, integrative review, narrative and/or bibliographic, reflective and theoretical studies and articles that did not address the aspects related to health care were excluded.

Three stages were structured for the organization of search strategies. To this end, the search strategy was used: "Men's health" AND "Care" in the Virtual Health Library (VHL) to find uncontrolled descriptors contained in the articles of interest. Then, there were combinations of controlled descriptors, obtained in the Health Sciences Descriptors (DeCS) and uncontrolled, obtained in the initial search, plus the Boolean operators "OR" and "AND". Finally, this strategy was adapted for each database (Table 1).

**Figure 1:**
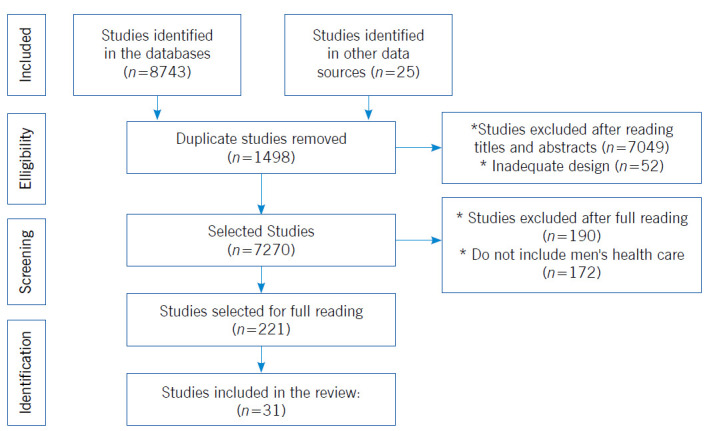
Flowchart according to Preferred Reporting Items for Systematic Reviews and Meta-Analyses (PRISMA)

The search and selection of the studies were carried out from September to December 2021, by two researchers who were authors of the study, independently, and the divergences were resolved by a third researcher. The following databases were used: MEDLINE/PubMed, CINAHL, LILACS, BDENF, Scopus, Web of Science and SciELO. For the management of the collected material, the Endnote was used. The selection of studies followed the recommendations of the Preferred Reporting Items for Systematic reviews and Meta-Analyses (PRISMA) method,^17^ represented in Figure 1.

For the development of the concept, we extracted from the empirical data: definition, concept, and antecedents, essential and consequent attributes. In this context, the defining essential attributes are configured in words or expressions that are used to structure the essential properties that determine the concept of interest, which makes it individualized from other existing analogous and/or approximate concepts. The antecedents and consequences of this concept are outlined as situations, events and/or incidents, which emerge a priori and a posteriori, in due order, the phenomenon of interest investigated, which results as a consequence, in this case, the health care of men. After this stage, the data were codified and allocated into analytical categories, in which the essential attributes of men's health care were composed of characteristics: Intrapersonal, psychological and behavioral related to masculinities, interpersonal, organizational and structural, ecological, ethnoracial, cross-cultural and transpersonal.

The analysis of the results of the review evidenced in the literature was descriptive, in which the synthesis of each study included in the review was presented, as well as comparisons between the studies. In addition, a thorough and exhaustive analysis of the direct relationship between the elements and the phenomenon investigated was carried out. 

## Results

The structuring of the development of the concept of health care for men is organized in the presentation of the scientific productions selected for the composition of the corpus of analysis, followed by the exposure of the use of terms related in the literature in different areas of knowledge, the delineation of the derivation/development of the concept from its essential attributes, the definition of the concept, the antecedents and consequences, the model case and the otherwise.

**Table 1:** Characterization of review articles according to title, year of publication and type of study.

Title in English	Year	Study type
1. Gender and health: the care of the man in debate^18^	2011	Cross-sectional
2. An Investigation of Health Management Perceptions and Wellness Behaviors in African American Males in Central Texas^19^	2018	Exploratory, qualitative
3. But if the man takes care of health, it is kind of paradoxical to work”: relationship between masculinities and health care for young men undergoing vocational training^20^	2018	Exploratory, qualitative
4. Male sex and health care: the experience of men in a health center^21^	2014	Descriptive, qualitative
5. Perceptions and behaviors of health care among elderly men)^22^	2012	Exploratory, qualitative
6. Experiences of recognition and care in the daily life of elderly rural men^23^	2018	Qualitative
7. Male health: the parallel between prevention and care in the light of the Planned Action Theory (PAT)^24^	2018	Exploratory, mixed
8. What Men Really Want: A Qualitative Investigation of Men's Health Needs From the Halton and St Helens Primary Care Trust Men's Health Promotion Project.^25^	2010	Qualitative
9. Insisting in the discourses related to self-care health practices in males^26^	2017	Qualitative, biographical
10. Migrants' Masculinity and Health. Taking Care for One's Health and Coping with Sickness of German Migrants in the US in 19th and Early 20th Centuries.^27^	2015	Historical, qualitative
11. Social representations of men about health and disease: contributions to care^28^	2015	Qualitative
12. Analysis of the health care of truck drivers^29^	2017	Quantitative, exploratory
13. Senses of Health from a Gender Perspective: a Study with Men from the City of Natal/RN^30^	2016	Qualitative
14. Perceptions of men's health from a relational perspective of gender, Brazil, 2014^31^	2017	Quantitative
15. Comprehensiveness of men's health care: a focus on quality of life^32^	2013	Descriptive, qualitative
16. Conception of health and self-care by the male population of a Basic Health Unit^33^	2015	Exploratory, qualitative
17. How do elderly men take care of their own health in primary care?^34^	2018	Qualitative
18. Care and life, family and work: multiple meanings of men's social representations of health^35^	2014	Qualitative
19. Male discourses on prevention and promotion of men's health^36^	2015	Exploratory, qualitative
20. Organizations in man's body: a nursing study on care^37^	2020	Qualitative
21. Health needs and masculinities: primary care for men^38^	2010	Qualitative
22. Conceptions and practices of care in the view of men^39^	2013	Qualitative, exploratory
23. Difficulties of male self-care: discourses of men who participate in an educational group for health.^40^	2020	Exploratory, qualitative
24. Health care for the male population in times of the National Policy for Integral Attention to Men's Health: what they say^41^	2015	Qualitative, exploratory
25. Discourse analysis on gender and health care of men admitted to a hospital^42^	2020	Exploratory, qualitative
26. Men's knowledge of body care: a cartographic study^43^	2020	Qualitative
27. Attitudes of care performed by hypertensive and diabetic men with respect to their health^44^	2012	Exploratory, qualitative
28. Analysis of the health care of truck drivers^45^	2017	Quantitative, exploratory
29. Masculinities and health practices: portraits of the research experience in Florianópolis/SC^46^	2017	Qualitative
30. Health care seeking among Mexican American men.^47^	2006	Qualitative
31. “Macho Men” and Preventive Health Care: Implications for Older Men in Different Social Classes.^48^	2011	Longitudinal

The identification of the use of terms related to the concept of health care of men present in the literature was organized by the appearance in the areas of knowledge, expressed in Table 1. 

**Table 2:** Identification of the use of terms related to men's health care in the literature in different areas of knowledge.

Area of knowledge	Use of terms related to men's health care
Nursing	[...] health care [...] self-care [...] health care [...] good nutrition, care, hygiene, life, physical activity, well-being and happiness [...].^35^
Medicine	[...] health care [...] preventive health care to the aggravations of chronic diseases [...].^21^
Psychology	[...] paradigmatic change of male perception in relation to their health care [...] knowledge about health care [...] male health care [...] health care [...] health care [...] new practices [...] lifestyles [...].^18^ [...] health care behaviors [...] self-care behavior [...] health care conditions [...] self-care [...] health care control [...] health care [...] maintaining health [...] good health [...].^22^
Public health	[...] health care for men [...] health promotion/prevention [...] recognition of the importance of health in their lives [...] health enhancers [...] personal organization to maintain healthy habits and/or the support provided by workplaces [...] health promotion practices [...] construction of gender models [...].^20^

When based on the survey, selection and dissection of the literature on the subject, submitting it to the derivation for the development of the concept, the essential attributes related to the health care of men were apprehended, represented in Table 3.

**Table 3:** Structure of the derivation of the concept development.

Dimensions/Aspects	Attributes	Characteristics of the attributes
Intrapersonal, psychological and behavioral related to masculinities	Acts of self-recognition as a caring being	Reception; affection; food; love; self-care; self-management and/or health management; monitoring; learning; self-care and/or self-care; attributions; activities; contraception; search; personal knowledge; control; deconstruction; decision; unveiling; elaboration; encouragement; choices; personal esteem; exercise; doing; enjoyment; imagination; intelligibility; intention; (inter)subjectivity; maintenance; birth; standardization; organization; particularization; promotion; prevention; principles; qualification; recognition; social and historical relationship; accountability; reproduction; satisfaction; sensitization; feeling; sexuality; signification; symbolic-cultural meanings about the masculine and the feminine; singularization; position-taking; valuation.
	States and processes that enhance the self-care of man	Autonomy; continuity; belief; expectation; improvement; mode; change; need; possibility; protagonism.
	Values and attitudes related to being a man that are linked to care	Ancestry; attention; self-assessment; self-confidence; self-esteem; self-respect; self-sufficiency; beauty; well-being; behavior; spiritualism; physical strength; history; intellectuality; masculinization; personality; psyche.
	Notions of man as a body	Biology; corporeality; body; life-generating body; cognition; physiology; functionality; body language; body medication; biological order.
	Unique experiences that lead men to take care of themselves	Illnesses; absences; accidents; damages; subjectivations; trajectories.
Personal	Care-seeking directions	Access; agency; help; support; approach; search; sharing; construction; involvement; gender; interaction; masculinity; procedures; search.
	Correspondence to socio-affective expectations	Orders; paternity; accomplishments; relationships; exchanges; valorization; bonding; zeal.
	Belonging and interactions in the socio-affective network	Friends; work environment; discourses; school; family members; drugs; intimacy; mothering; guidance; paternity; paternity; educational processes; health professionals; protection; sex; reciprocity; support network; symbolism; systems; society; solidarity; therapeutics; neighbors.
Macrosocial	Sociohistorical processes	Social agencies; social behavior; social coexistence; right (s); economy; social esteem; social structure; generation; history; life stories; ideology; ideologies; ways of living; social movements; objectification; occupation; organizations; power; politicization; positions; social representation; reproduction of social life.
	Collective interactions	Scenarios; cycles and circuits of life; collectivity; communication; community; gender construction; contexts; social determination; distribution of power; social identity; comprehensiveness; freedom; machismo; masculinities; naturalness; belonging; politics; risk; collective senses; health situation; work.
Ecological	Ecosystem connections and actions	Environment; animals; agriculture; community; customs; ecology; spaces; locality; world; nationality; home healing methods; nature; organism; predisposition; preservation; regionalization; land; territorialization; vegetation.
Ethnic-racial	Ethnic agreement	Afrocentrism; proximity to a professional of color - between peers; trust and ethnic relationship; ethnic religiosity; ethnic traditions.
Cross-cultural	Incorporation and cultural expression	Acculturation; cultural events; human grouping; appropriation; cultural knowledge; customs; intimate convictions; cultural beliefs; cultural teachings; habits; cultural heritages; cultural hierarchies; mourning; magic; cultural norms; cultural patterns; regionalisms; cultural signs; common sense; cultural symbols; superstition; cultural traits; uses; cultural values.
Transpersonal	Transcendence	Consciousness; cosmos; spiritual belief; spirituality; balance; existence; faith; happiness; phenomena; humanism; death/dying; passage; positivity; religiosity; health; safety; transformation; temporality; value; life.

In order to illustrate the operability of the applicability of the concept of men's health care, a *model case* was developed, described below: [...] "Adult man has observed his body, his mind and spirit daily, which made it possible to recognize a drop in his income at work. When experiencing pain and changes in sexual dynamics, he talks to his partner; he seeks to consume foods that he believes can increase potency, indicated by friends. He reflects on what occurs to him and seeks to recognize the place of this pain, and the reasons for its occurrence, later, he tries to change the pace of his activities, adopts physical exercises and relaxation; he tries to think of positive situations. He uses the references of care experienced in the past, seeks guidance from significant people, family, friends, co-workers and neighbors; he adopts integrative and complementary practices, seeks assistance in traditional spaces and in health services. He recognizes himself as vulnerable, external to his desires, fears, and concerns and expresses his feelings and emotions. He reveals the tensions between his state and the pressures of hegemonic masculinities. When feeling afraid of becoming ill, he resorts to his socio-affective, environmental, bioenergetic, ethnic/racial, cultural, spiritual and/or religious network, and allows himself to be cared for by others to face the adverse situation and reintegrate into his daily activities that make up his sociohistory”. 

As a way to illustrate the non-operability of the concept of men's health care, the opposite *is* presented, as follows: [...] “Adult man realized that he has not been yielding at work as before, he feels constant pain but takes medication on his own or drinks a beer. He believes that this discomfort will pass, because he thinks he is strong enough to overcome this situation and continue working in the same way. When he realizes that tiredness affects his sexual activity, he avoids intimate encounters and touching this aspect with his partner. Sometimes he is afraid of getting sick, he feels irritated and he feels confident not to cry, he thinks about seeking help, but soon gives up thinking about it he can solve this situation alone and goes back to work”.

The structuring of antecedents and consequences for the health care of men are represented from the qualification as harmonious and disharmonious, when considering their characteristics in relation to health care. 

### Background

The antecedents of the concept were structured in dimensions: intrapersonal (related to psychological, behavioral aspects related to masculinities), interpersonal, macrosocial, ecological, ethnic-racial, cross-cultural and transpersonal, which relate to the interactions described below: 

*Intrapersonal (psychological and behavioral related to masculinities):* Illness; autonomy and independence; cycles and/or phases of life; personal beliefs; male beliefs; food and body culture; diseases; male identity; normative models; intelligible practices; performances; subsistence; male universe; force vectors; experiences.

*Interpersonal (socio-affective interaction)*: Discursive fields; affective determinants; medical discourses; femininity; female historical-cultural reference; medical guidance; therapeutic plans; marital, family, maternal, paternal and socio-affective relations; references of care; sexualities; female universe.

Macrosocial (interaction with structures of society): Accesses - diagnostic tests, internet, health services; social environment; provided by institutions; conjuncture; daily life; contexts - social and life; social constructions - gender - masculinities; social class; culture; social determinants; city dynamics; factors - generational, historical, political, risk; communication - cellular telephony; social imaginary; social metaphors; health care models; world of work - conditions, locations, value; organization of health services; social organization; health plan; family provision; racial relations; wealth; economic situation; social *status*; urbanization.

*Ecological (interaction with the environment):* Animal and plant coexistence; emigration and migratory flows; territorial and regional insertion; geographical location; nationality; agricultural, environmental, ecological and ethnic relations.

*Ethnic-racial:* Ethnic-centered experiences; ethnic teachings; ethnic acculturation.

*Cross-cultural (interaction with culture):* Adoption and influence of local customs and popular knowledge.

*Transpersonal:* Adoption of beliefs and philosophies; desires around personal fulfillment; ancestral, spatial, spiritual, faith and religious influences; ways of existing, seeing care, health and living life; ritual practices.

### Consequent

The consequent of the concept were structured in dimensions: intrapersonal (related to psychological, behavioral aspects related to masculinities), interpersonal, macrosocial, ecological, ethnic-racial, cross-cultural and transpersonal, described below:

*Intrapersonal:* Healthy and balanced eating; abandonment of habits harmful to health and/or addictions; adoption and directing efforts to promote a healthy lifestyle and habits, preventive care with physical and mental health, aesthetics, relaxation, leisure, daily measures to control diseases, improvement of sleep pattern; learning, perception and concern with health care; self-examination; evaluation of signs and symptoms; knowledge of the body; control in food consumption, weight, metabolic levels, blood perfusion; moderate consumption of alcoholic beverages; dedication to work and the family; decrease in exposure to the risks of greater health problems, the emergence of diseases and aggravation of others already existing; elaboration of individualized forms of care; adequate coping with the effects and treatments of diseases; emission of healthy behaviors; positive coping with the disease and/or health problems; hydration; hygiene; independence for self-care; management of intrapersonal barriers to care; manifestation of emotions and feelings; maintenance of the practice of positive attitudes towards oneself, life, health status, good functioning of the body and continuity of care and healthy longevity; changes in health behaviors; objectification of care, health and life; personal organization and care routines; health promotion, physical, psychosocial and spiritual well-being and health prudence; recognition of the importance of health care; rest; subversion; personal and symbolic appreciation; zeal.

*Interpersonal:* Adherence and regular monitoring of consultations and/or interventions and/or prescriptions and/or treatments and/or health-disease-aggravation therapies, implemented by professionals in health services; Adherence to available family support; Adoption of care with the home environment; Support received and provided to loved ones/family members; Care of the other; Care for sexual and reproductive health; Child and/or child care; Being with the other; Establishing relationships with and between people; Affective involvement and/or manifestation of affection; Establishment and strengthening of trust and family and socio-affective bond; Interaction with health professionals; Immunization; Modeling of good behavior with others; Practice of physical activity and/or physical exercises; Obtaining referrals from health services; Participation in activities offered in the health service (individual and collective); Preservation of marriage and harmony of the home and health habits arising from conjugality; Medical search for diagnosis; Conducting diagnostic tests; Recreation and/or socialization with friends; Overcoming weaknesses caused by widowhood; Use of medications according to medical prescription and/or safe recommendation; Use of primary health services.

*Macrosocial:* Access to attractions in the community; support received in the workplace; search for help from others; search for improvement in quality of life; sharing with the social other; collective coexistence; work performance; willingness to obtain social support; engagement in intellectual activities; involvement in social projects; exercise of good coexistence and company; strengthening with the social network; engagement in labor and union activities; structuring of modes for care, health and life; establishment of levels of care and health care; management of responsibilities towards the family; management of barriers imposed by daily, by work, by the health system and by economic/financial difficulties; maintenance of functional and work capacity; improvement of social skills; motivation to attend public spaces; participation of group activities in the community and/or educational groups; promotion of future reduction of spending on health; promotion of social and work well-being; provision of material resources for care to occur; preservation of the workforce; reduction of the pace of daily activities and work; overcoming patriarchal values and gender barriers, masculinities and other social character; use of informational support in access to guidance and instruments arising from social incentive; healthy life.

*Ecological:* Agricultural cultivation; ecosophy; establishment and/or closer ties with the environment/earth; environmental preservation.

*Ethnic-racial:* Racial self-affirmation; good ethnic coexistence; establishment of mechanisms for overcoming and combating racism and racial discrimination; strengthening of ethnic relationships; valuing knowledge, cultures and ethnic traditions.

*Transcultural (interaction with culture):* Ancient knowledge transmitted from generation to generation; search for traditional knowledge; preservation of family traditions; overcoming cultural barriers; use of teas and products and/or natural and/or home remedies from traditional and/or ancestral popular knowledge.

*Transpersonal:* Achievement of happiness; accumulation and/or acquisition of life experiences; well-being; search for help, assistance and/or spiritual and life healing; search for balance; going to church; participating in religious groups; promotion of spiritual well-being.

### Concept definition

From the identification of the attributes of the concept of “men's health care”, the stage of conceptual definition was carried out. The concept developed showed that the health care of men is based on previous references and is structured in male and female learning of gender, which outline attitudes, behaviors, stereotypes and practices that demarcate impressions of the place of man in the ways of being, of seeing himself, health, care, environment and life: [...] *"The health care of men is a set of conceptions, attitudes and practices, which is structured individually and/or collectively in the intrapersonal, behavioral, interpersonal, macrosocial, ecological, cross-cultural, ethnocultural and transpersonal dimensions in a given socio-cultural and territorial inscription. This care is directed both individually and collectively to men and by themselves, based on the beliefs and expectations about the male being in the world. In the individual sphere and experience, care is manifested anchored in the notions of the productive and sexual body, in an intersubjective and psycho-emotional way, which involves values, self-recognition of the need to take care of oneself, singular experiences, search for personal fulfillment, ethnic agreement and transcendence. In the collective sphere, interpersonally care is manifested in interactions with its socio-affective network; community, environmental and culturally emerges and is expressed under the influence of the social construction of masculinities and femininities, of ecosystemic and socio-historical experiences”.*

## Discussion

When raising the essential attributes of the concept of men's health care, the expressiveness of existing phenomena was observed, which had different ramifications and origins, which led us to outline structures that were based on the dimensions of men's being and existence in a given socio-historical, cultural, gender and masculinities context, sexual identities, territory, schooling, age and generation, race/color/ethnicity, the world of work, daily and complex life and social class.^18^^-^^43^ Thus, the following dimensions were recognized: intrapersonal, behavioral, interpersonal, organizational, structural, ecological and transpersonal in a given socio-cultural and territorial inscription.

To develop the concept from the identification of essential attributes, it was noted in the material that there was a greater centrality and frequency of appearance of findings related to men's health care with regard to the set of actions, attitudes, characteristics, conceptions, components, state, motivations, orientations, perceptions, positions, feelings and experiences arising from the identity constructions of men. In addition, they were also linked to the principles, values, subjectivities, imaginary and intelligibilities that strongly guided the construction of habits and lifestyle in relation to health care.^21^^-^^26^

The health care of men reserves great centrality in the individual sphere of the subjects, which has a significant approximation with the hegemonic aspects of masculinities, when gender norms based on patriarchy and machismo are printed. Such findings may result in harm to the self-care of health by influencing the adoption of isolated, unsafe care measures, as seen in the context of male self-medicalization, the removal of this public from health services, resistance to adherence to health therapies and prevention strategies. 

Although the studies were developed between the 1990s and 2000s, men of different ages were investigated, and even the most current studies, there was a marked nuance for the exercise of biophysiological care, corporeality.^20^^,^^34^^-^^38^^,^^43^ There was also a significant concentration in relation to body archetypes associated with aesthetics, food coordinated by nutritional supervision, body and intimate hygiene care, care that emerges from an intense relationship with the disease, which makes it possible to light up the care denominated as preventive, medical, therapeutic, diagnostic and assistance.^20^^,^^34^^-^^38^^,^^43^ Even considering this centrality in the studies apprehended, the development of the concept of men's health care is not limited to this dimension, since, from the diversity of constructions of existing masculinities, new dimensions were evidenced in care, which reveal its character of progressivity, temporality and transition in the individual and collective dynamics of the male audience in its territories and distinct cultures.^18^^-^^19^^,^^21^^,^^23^^,^^25^^,^^27^^,^^34^

Regarding the dimension of interpersonal essential attributes, we observe the presence of communication phenomena, the relationship that men establish with and among people who are in their cycle and/or socio-affective network.^18^^-^^22^^,^^35^^,^^38^ These attributes are also associated with existing psychosocial relationships and established from symbolic interactions, which can be noticed in the access and/or search for health services, for help and/or support provided or received, such as the relations of exchanges, approximations, sharing, and the action of phenomena that occur in the places of belonging and movement of men, such as the family environment, workplaces, integration with friends and neighbors, experiences arising from intimate and sexual relationships, and health services.^18^^-^^22^^,^^35^^,^^38^ In addition, the influence of support systems, medical discourses, therapeutic plans that are instituted, by popular knowledge passed on by the elders, and finally, school spaces and educational training processes and society stands out.^35^^,^^38^^-^^42^

The essential macrosocial attributes are related to phenomena arising from occupation, family, socioeconomic level, culture, values and social ideologies and evolutionary relationships of intra and interpersonal character of society. Men's health care is closely linked to the dynamics of the world of work, social class, mobility, exchanges, social conflicts, as well as emerging from ways of life, everyday life and complexity. In this sense, the attributes reveal the emergence of processes such as assemblages, cultural influences, representations, life stories, objectification, organization, daily life, ethics, the health-disease process, health promotion, quality of life, social responses, routines, experiences and vulnerabilities.^19^^-^^23^ When considering the framework of these attributes to the organizational and structural dimension, essential implications are promoted for the recognition of male specificities in their *locus* of social and political inscription.^35^^,^^38^^-^^43^ From the surveys of the constituent elements of the attributes, great emphasis was observed on the relationships of men with their professional work, the positions occupied in society, the relationship of *status* and responsibilities required and the way these elements influence the health care of men^44^^-^^47^ in the most varied communities, whether urban, or peasant, rural and riverside,^23^ as located in the studies.

When trying to identify essential attributes and frame them in ecological dimensions, in which the presence of a health care interconnected to the habitat, the ecological niche and the intimacy of men with the maintenance of the ecosystem was observed, specific phenomena were glimpsed. In this way, the preservation of local customs, the position of being in community, ecosophy, the relationship with the physical and social, animal and vegetable environment, as well as the presence of the interrelationship with space, the world, nature, organisms and the earth, were identified.^19^^,^^25^^,^^27^ On these ecological attributes, it should be noted that they were recognized especially among men who live in direct contact with the land, planting, as well as those who had traditional reference bases for the relationship with the environment and ecology, to the point of considering these elements as essential components of health care.^19^^,^^25^^,^^27^

It was also noted the incorporation of attributes related to ethnic and cross-cultural dimensions, seized in studies whose male population investigated was permeated by the influence of structural markers of age and generation - elderly men, territory - rural, riverside and peasant men and race/color - black men.^19^^,(^^23^^-^^27^^),^^37^^,^^43^^,^^48^ It should be noted that the selected studies did not deepen the discussions on ethnicity. Therefore, other races were not evidenced, such as indigenous men, which reveals a gap to be explored and contemplated.^49^^-^^52^ In addition, the phenomena present in the energy, physical, emotional, mental and spiritual fields, also through the interface with the spirituality and religious knowledge of men, are part of the essential transpersonal attributes of the concept of health care for men.^24^^,^^26^^,^^30^^-^^31^ Thus, the attributes are the passage, transformation, religious beliefs, balance, being alive, existence, faith, happiness, positivity and human value. It also includes phenomena, health, safety, temporality, consciousness and the cosmos.^(53)^

## Conclusion

The concept of men's health care involved the following dimensions/aspects: intrapersonal, psychological and behavioral related to masculinities, interpersonal, macro-social, ecological, ethnic-racial, cross-cultural and transpersonal. In addition, the impact of this study is based on the advancement of scientific knowledge on the subject and the potential for feasibility in generating contributions to health science and practice, whether from an epistemological and conceptual perspective, or to practice from the reformulation of public policies, orientation of the service and health care and construction of care technologies. The concept analyzed can be useful to support the practice directed to the health care of men in the various scenarios of action, in the field of nursing and health, strengthening the structuring of instruments for clinical care, teaching, research and development of public policies for men, considering the antecedents, attributes and consequences of the care of this population.
